# Pacemaker-induced atrial fibrillation reconsidered—associations with different pacing sites and prevention approaches

**DOI:** 10.3389/fcvm.2024.1412283

**Published:** 2024-06-18

**Authors:** Fabienne Kreimer, Michael Gotzmann

**Affiliations:** Department of Cardiology and Rhythmology, St Josef Hospital Bochum, University Hospital of the Ruhr University Bochum, Bochum, Germany

**Keywords:** atrial fibrillation, pacemaker, pacing sites, pacing modes, ventricular, atrial

## Abstract

The incidence of atrial fibrillation (AF) is significantly higher in patients with pacemakers than in the general population, which could be due to patient characteristics and the diagnostic tool of the pacemaker in detecting atrial high-rate episodes and subclinical AF, but also to the pacemaker itself providing AF-promoting conditions. It is well known that high ventricular pacemaker burden increases the likelihood of AF occurrence. However, the sites of atrial and ventricular pacing may also influence the risk for AF. The conventional sites for atrial and ventricular pacing are in the right atrial appendage and in the right ventricular apex. However, growing evidence suggests that alternative pacing sites may be superior for the prevention of AF. Bachmann bundle pacing, for example, promotes interatrial excitation conduction, resulting in atrial synchronicity and a shorter total atrial activation time, which may be preventive for the occurrence of AF. Moreover, in recent years, new ventricular pacing sites have come into focus with His bundle and left bundle branch pacing. In addition to the hemodynamic and electrophysiological cardiac benefits, these new options may also offer benefits in the prevention of AF. This review provides an overview of pacing-induced AF mechanisms and the association with different pacing sites, as well as approaches for prevention of pacing-induced AF, highlighting different sites and modes of atrial pacing and the newer sites of ventricular pacing.

## 1 Introduction

The incidence of atrial fibrillation (AF) is several-fold higher in patients with pacemakers than in the general population without pacemakers (1). The annual incidence of AF is at least 5% after pacemaker implantation, and the mean lifetime cumulative incidence can be estimated to be approximately 30%–40% (1).

This increased incidence can be explained by three factors: (1) Patients receiving pacemakers typically have advanced age and age-related degenerative changes, and therefore also often have a higher burden of cardiovascular disease than the general population (2). (2) Incidence rates may be influenced by the detection of atrial high-rate episodes and subclinical AF, as part of the diagnostic tools of a pacemaker (3, 4). (3) Pacing may accelerate the progression of pre-existing AF.

Both atrial and ventricular pacing sites and different pacing modes influence the development and maintenance of AF (5). This article provides an overview of the contribution of different pacing sites and modes to the prevention of AF. As conventional ventricular pacing is well known to increase the likelihood of AF, the review focuses on atrial pacing sites and modes as well as more recent ventricular pacing sites such as His bundle pacing and left bundle branch pacing.

## 2 Pathophysiology and mechanisms of pacemaker-induced atrial fibrillation

Due to electrophysiologic and hemodynamic alterations as well as atrial and ventricular desynchrony pacing itself provides promoting conditions that may trigger and precipitate AF. Mechanisms for pacemaker-associated AF include cardiac electrical and structural remodeling, inflammation, and autonomic nervous disorder (6).

One of the most important mechanisms contributing to atrial fibrillation after pacemaker implantation is electromechanical remodeling of the atria (6). Chronic pacing, particularly in the right ventricle, can lead to asynchronous contraction patterns that can result in abnormal changes in ion channel function and electrical coupling between cardiac myocytes (6). Constant pacing alters normal electrical excitation propagation and causes regions of delayed or early activation, which may favor the development of reentrant circuits and thus the development and maintenance of AF (6). In addition, the implantation process can cause local inflammation and subsequent fibrosis in the atrial tissue (7). This fibrosis alters the structural and electrical properties of the atria and creates a substrate for atrial arrhythmias (7).

Pacemaker implantation can affect the autonomic nervous system, particularly by altering the balance between sympathetic and parasympathetic tone (6). Increased sympathetic activity or decreased parasympathetic activity can increase the excitability of the atria and shorten the refractory period, facilitating the development of AF (6).

Alterations in cardiac hemodynamics after implantation can also contribute to AF (8, 9). For example, right ventricular pacing can lead to decreased cardiac output, tricuspid and mitral valve regurgitation and increased atrial pressure, which stretches atrial myocytes and promotes electrical instability (10). Loss of atrioventricular synchrony can also lead to increased atrial pressure and the development of AF (10).

## 3 Atrial pacing

**Figure 1:**
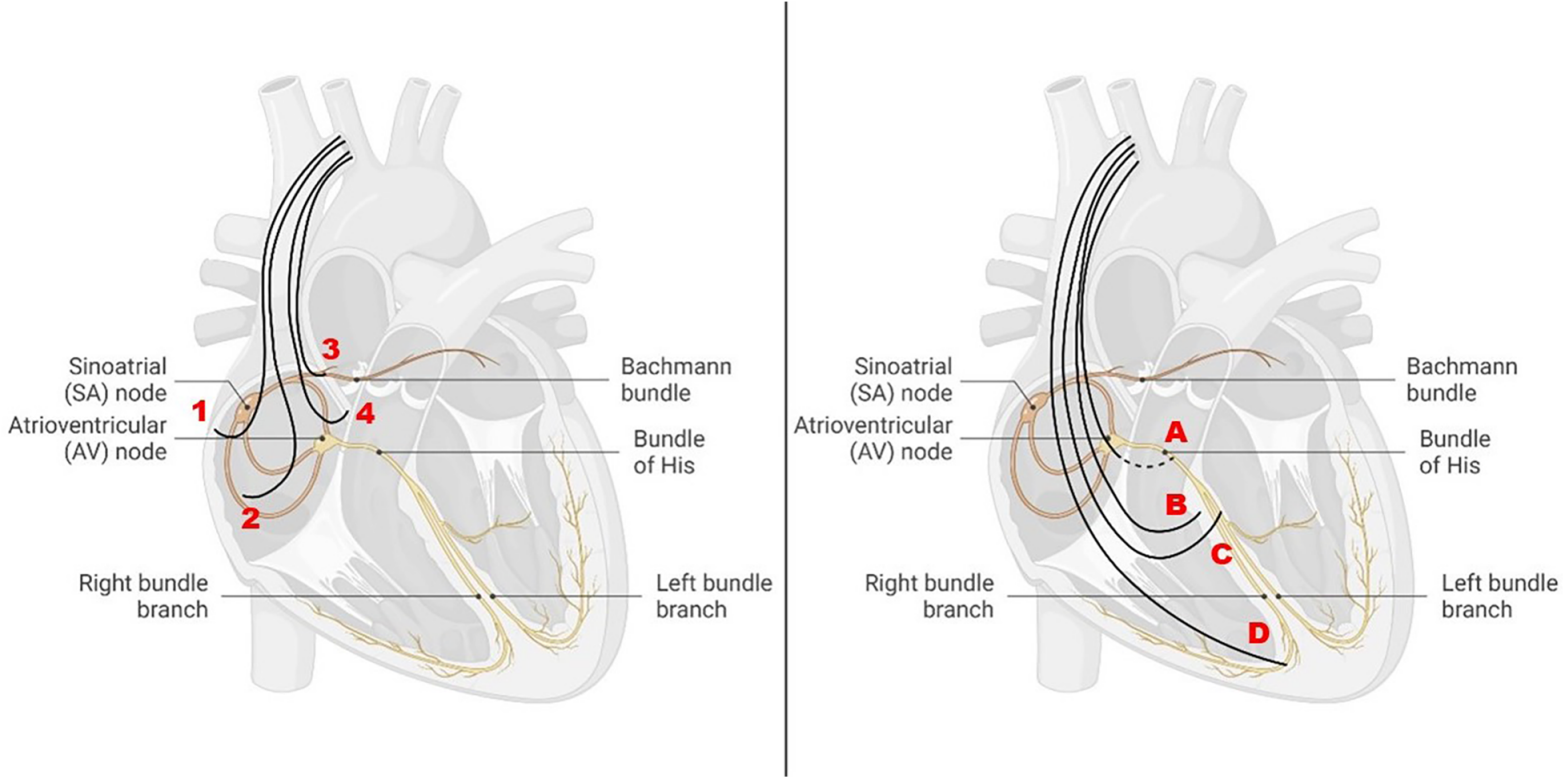
Different atrial and ventricular pacing sites in relation to the cardiac conduction system. Left: atrial pacing sites at the right atrial appendage (1), at the right lateral wall (2), at the Bachmann bundle (3), and at the interatrial septum (4). Right: ventricular pacing sites in the region of the His bundle (**A**), at the high septum (**B**), at the left bundle branch (**C**), and at the right ventricular apex (**D**).

In healthy individuals, physiological cardiac excitation begins in the sinus node near the entrance of the superior vena cava into the right atrium, extends therefrom via the right atrium, and subsequently excites the left atrium, predominantly via the Bachmann bundle (BB) as an interatrial junction (Figure 1).

Sinus node disease (SND) and interatrial conduction delays are associated with AF, which may be explained by the widening of the time frame in which atrial ectopy can trigger AF. AF-promoting factors include dispersion of atrial refractoriness, prolonged interatrial conduction, as well as desynchronized atrial excitation, reflected by prolonged total atrial activation time (11–14). For this reason, it seems feasible that atrial pacing is optimized when the dispersion of atrial refractoriness is as minimal as possible, interatrial conduction is as rapid as possible, and atrial activation is maximally synchronized to minimize the risk for AF.

Electrophysiological alterations that can accompany atrial pacing involve both P-wave duration and morphology, atrial synchrony, and AV conduction.

In the following, different atrial pacing sites and their hemodynamic as well as electrophysiological effects are presented and discussed (Figure 1 left) (Table 1). In addition, the risk for AF associated with the different pacing sites will be highlighted.

**Table 1:** Different atrial pacing sites and their advantages and disadvantages.

Atrial pacing site	Advantages	Disadvantages
Right atrial appendage	• Simple, even easily achievable for the inexperienced • Stable lead position with low displacement rates • Good sensing and stimulation values	• Negative electrophysiological hemodynamic effects: prolonged P-wave duration, prolongation of total atrial activation time, prolongation of intra- and interatrial conduction • Evidence for increased incidence of AF, increased recurrence of AF, increased number of AF episodes and more frequently progression of AF
Right atrial free lateral wall	• Good sensing and stimulation values	• Highest risk of perforation due to a thin wall at this position with complications like pericardial effusion and tamponade
Interatrial septum Low interatrial septum High interatrial septum	• Positive electrophysiological and hemodynamic effects: improved global and regional atrial mechanical function and synchronized interatrial electromechanical contraction, lower P-wave duration, decreased atrioventricular interval, prevention of LA enlargement • Evidence for reduced AF episodes and burden, fewer symptomatic AF epsiodes • (Lead-related complications like right atrial appendage pacing)	• No benefits in the prevention of persistent or permanent AF • Low interatrial septum: difficult positioning of the lead, risk of displacement is higher, poor sensing and stimulation values
Bachmann bundle	• Good sensing and stimulation values • Stable lead position with low displacement rates • Positive electrophysiological and hemodynamic effects: improved interatrial conduction, atrial synchrony, lower P-wave duration, improved atrial mechanical function, decreased atrioventricular conduction time • May reduce the ventricular pacing burden • Evidence for decreased AF inducibility, reduced new-onset AF, reduced AF recurrence, reduced AF progression	• Challenging lead positioning in difficult left atrial anatomy
Right atrial multisite	• Positive electrophysiological and hemodynamic effects: improved mechanical atrial function, prevention of LA enlargement • Low evidence: prolonged time to recurrent AF	• No reduction of persistent or permanent AF • High risk of displacement, higher implantation effort, more complications
Biatrial (coronary sinus + right atrial)	• Positive electrophysiological and hemodynamic effects: improved mechanical atrial function, reduced atrial conduction delay, prevention of LA enlargement, improved atrioventricular synchrony	• No reduction of persistent or permanent AF • High risk of displacement, higher implantation effort, more complications

### 3.1 Different atrial pacing sites, their hemodynamic and electrophysiological effects, and the risk of atrial fibrillation

#### 3.1.1 Right atrial appendage pacing

**Figure 2:**
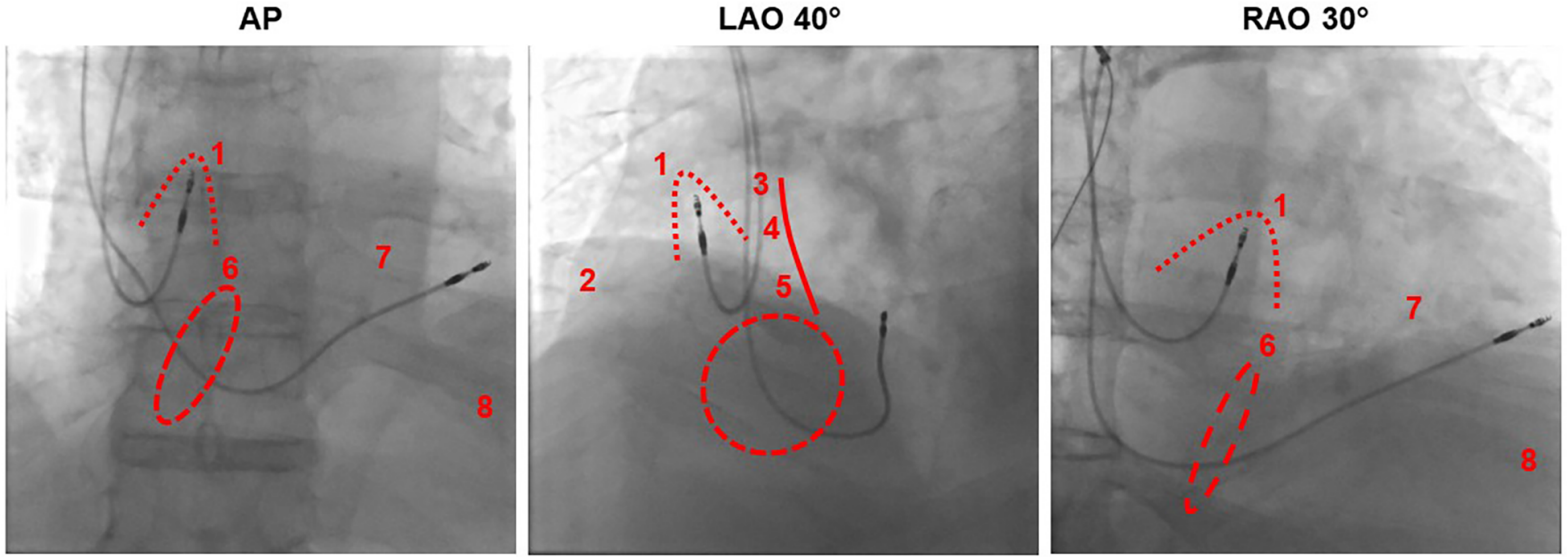
Fluoroscopic imaging of atrial lead positioning in the right atrial appendage from different projections in relation to different pacing sites. AP, anterior-posterior; LAO, left anterior oblique; RAO, right anterior oblique. Dotted line: right atrial appendage, dashed line: tricuspid valve, solid line: atrial septum. (1) Right atrial appendage, (2) right atrial free wall, (3) Bachmann bundle, (4) high septal, (5) low septal, (6) his bundle near the tricuspid anulus, (7) right ventricular high septal, (8) right ventricular apical.

The positioning of the right atrial electrode in the right atrial appendage (RAA) is usually the first choice for implantation as it is relatively simple, even for an inexperienced clinician, and there is a low dislocation rate in this position. Another advantage is that good sensing and stimulation values are usually achieved here. Furthermore, the correct position of the electrode can be fluoroscopically verified during implantation (Figure 2).

**Figure 3:**
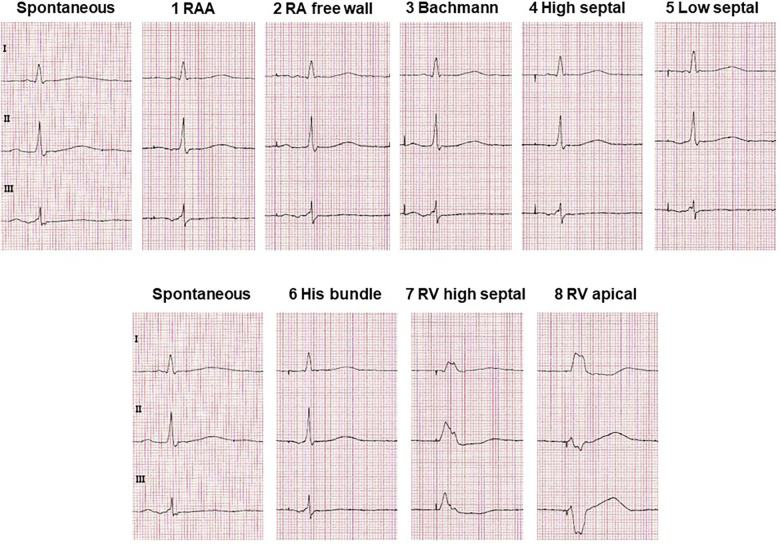
Typical ECG alterations in leads I, II and III with pacing at different atrial and ventricular lead positions compared with non-paced sinus ECG. Excerpts from ECGs are shown which were recorded at a rate of 50 mm/s and a voltage of 10 mm/mV.

However, there have been observations that placement of the atrial electrode in the RAA may be inferior to other atrial pacing sites in terms of optimal excitation conduction and hemodynamic effects: Conventional stimulation of the RAA in the presence of interatrial conduction delays results in a pronounced latency with prolonged P-wave duration and reduced amplitude, whereas P-wave morphology is similar to sinus rhythm (15) (Figure 3). Furthermore, conventional placement of the atrial electrode in the RAA results in prolongation of the total activation time of both atria (16). Electrophysiological alterations accompanied by RAA pacing also led to increased AF inducibility. In a study of patients with paroxysmal AF, AF was easily induced by extrastimuli during RAA pacing (17). In contrast, BB, right posterior interatrial septum (IAS), and distal coronary sinus pacing appeared to be more effective in preventing AF, even more than multisite and biatrial pacing (17).

The PASTA trial was a randomized prospective study examining the effect of different atrial lead placements on the incidence of AF (18). The analysis included 142 patients with SND who were assigned to the pacing site groups of free right atrial wall, RAA, coronary sinus ostium, or dual-site right atrial pacing, which included simultaneous RAA and coronary sinus ostium pacing (18). There was no significant difference among the four groups in the occurrence of AF within 2 years (18). AF detection rates were 36%, 38%, 32%, and 48% in the free right atrial wall, RAA, coronary sinus ostium, or dual-site right atrial pacing group, respectively (18). RAA was therefore not inferior in terms of the endpoint incidence of AF.

However, in patients with SND and AF before implantation, RAA pacing may cause significant intraatrial conduction disturbance and, consequently, increase the risk of AF recurrence, especially in patients with a prolonged paced P wave (19). The risk for AF recurrence is significantly higher with a paced P-wave duration >130 ms (19). Thus, when implanting the atrial lead, looking at the paced P-wave may be helpful in deciding whether the RAA should be favored as a pacing site or an alternative one.

Moreover, a meta-analysis by Zhang et al., which included 1,146 patients with a DDD pacemaker, demonstrated that RAA pacing was inferior to IAS pacing in reducing the number of AF episodes (20).

A progression from paroxysmal to persistent or permanent AF may reflect atrial remodeling. Therefore, AF progression also represents an important end point of studies comparing different atrial pacing sites. The randomized, controlled, prospective EPASS study investigated 97 patients with paroxysmal AF and RAA or IAS pacing (21). The primary endpoint of the study was time to onset of persistent or permanent AF within a 2-year follow-up period (21). After a mean follow-up time of 15 ± 7 months, 11 (16.6%) patients in the study group met the primary end point: 2 patients in the IAS vs. 9 patients in the RAA group, which was significant (21). RAA pacing was thus inferior in preventing progression to persistent or permanent AF. However, the meta-analysis by Zhang et al. demonstrated that the occurrence of permanent AF was not significantly more frequent in RAA pacing compared with IAS pacing (20).

Overall, electrophysiological studies suggest an increased risk of inducing AF with RAA stimulation, whereas evidence from clinical trials is inconsistent.

#### 3.1.2 Right atrial lateral wall pacing

The lateral right atrial free wall is not a preferred site of atrial lead placement for clinicians, due to the risk of perforation of the right atrial lead in the very thin wall of the right atrium. However, Pernollet et al. recently demonstrated that 4 years after pacemaker implantation, AF occurred in 26.6% of the RAA group and only 9.7% of the lateral group (22). Pacing at the right atrial lateral wall was associated with a significantly lower incidence of AF compared with conventional RAA pacing (22). As described above, the PASTA trial did not detect a statistically significant difference in the incidence of AF between the four groups, including lateral right atrial wall, RAA, coronary sinus ostium, or dual-site right atrial pacing, after 24 months of follow-up (18). Thus, superiority of lateral right atrial pacing compared with conventional RAA pacing has not been clearly demonstrated to date.

#### 3.1.3 Interatrial septum pacing

IAS pacing is supposed to be beneficial over RAA pacing in improving interatrial excitation conduction and resembling the physiological excitation process to a greater extent, as reflected electrocardiographically by, e.g., a lower P-wave duration (17) (Figure 3). IAS pacing in patients with paroxysmal AF reduces the degree of P-wave dispersion and the occurrence of atrial late potentials in P-wave signal-averaged electrocardiography compared with RAA pacing (23). IAS stimulation has previously been demonstrated to not only result in shorter atrial conduction time and P-wave duration, but also to be beneficial for atrial synchronization (24).

Moreover, an influence of the atrial pacing site on left atrial cardiac output were reported, as left atrial ejection fraction and active left atrial emptying fraction were higher during IAS pacing compared with conventional RAA pacing (25). In addition, atrial velocity at both the right atrial free wall and the left atrial septum was higher during IAS pacing compared with RAA pacing (25).

Most studies on IAS pacing included patients with paroxysmal AF, comparing it to RAA pacing as an established pacing site (17, 20, 23, 26–29). It was observed that paroxysmal AF episodes can be reduced significantly more often with IAS pacing than with RAA pacing (27). Moreover, paroxysmal AF burden was significantly lower in patients with IAS pacing compared with RAA pacing (27). These findings were also confirmed in a meta-analysis by Shali et al. examining randomized-controlled trials of IAS vs. conventional RAA pacing (29). A total of 1,245 patients with paroxysmal AF were included. IAS pacing was associated with significantly lower device-detected AF burden and AF frequency (29). The likelihood of lead-related complications and the combined rate of major adverse events were similar in both groups (29). Hence, it can be concluded that IAS pacing appears to reduce device-detected AF burden and AF frequency while carrying a similar risk of electrode-related complications as RAA pacing (29).

In contrast, other studies were less positive and did not detect different rates of AF-free survival, device-detected AF burden and frequency between IAS and RAA pacing (26, 28).

There was no additional expected benefit in the prevention of persistent and permanent AF: Septal pacing failed to prolong survival without persistent/permanent AF compared with RAA pacing (29). Similarly, the meta-analysis by Zhang et al. concluded that although IAS pacing was superior to RAA pacing in terms of reduction of AF episodes, AF burden, and P-wave duration, there was no significant difference in the occurrence or recurrence of longer-lasting AF (20).

In conclusion, IAS stimulation is safe and as well tolerated as RAA stimulation. Although IAS pacing cannot prevent the occurrence of prolonged AF or the recurrence of AF, it has the advantage of improving not only interatrial excitation conduction but also reducing AF burden.

#### 3.1.4 Low interatrial septal pacing

Positioning of the right atrial lead in the inferior IAS is difficult and is associated with the risk of dislocation. In the past, low IAS pacing has been demonstrated to significantly improve global and regional atrial mechanical function and synchronized interatrial electromechanical contraction compared with RAA pacing (25). In addition, compared with RAA pacing, low IAS pacing shortened the atrioventricular interval in SND patients with or without first-degree atrioventricular block and prevented left atrial enlargement in the long term (30).

Minamiguchi et al. analyzed 95 patients with SND, who received low IAS pacing or RAA pacing, for association with AF (31). During 1-year follow-up, 19.0% of patients without pre-existing AF in the RAA group developed incident AF, but only 5.9% of the low IAS group (31). However, because of the modest absolute number, the difference was not significant. Among patients with a history of AF, 22.0% of the RAA group developed persistent AF, but none of the low IAS group (31). Moreover, no postoperative complications related to low IAS pacing occurred (31). The EPASS trial reinforced these findings by demonstrating that low IAS pacing is superior to RAA pacing in preventing progression to persistent or permanent AF (21). However, similar to IAS pacing in general, the data situation is inconsistent, because there is also evidence that low IAS pacing compared with RAA pacing cannot prevent the development of persistent AF and is therefore not superior to RAA pacing (32).

#### 3.1.5 High interatrial septal pacing

Hakacova et al. compared high IAS pacing with IAS pacing in 43 patients with paroxysmal AF and an indication for a DDD pacemaker (33). Neither the number of mode-switching episodes nor AF burden differed significantly between the groups, although there was a trend toward less AF with IAS pacing (33). Furthermore, there were no differences in thresholds, detection, or electrode impedance, and electrode parameters also remained stable over time (33). Complications related to the electrodes did not occur. Thus, the authors concluded that implantation of an atrial-active fixation electrode at the atrial septum is safe and feasible, but no significant difference between septal pacing and high-atrial pacing was found based on the end points of AF duration and number of AF episodes (33). These results may suggest that the exact location of the atrial lead at the IAS is less important for a better outcome than the fact that IAS pacing may be superior to other pacing sites. However, it is important to note that there is a lack of studies, and evidence for an advantage of IAS pacing over RAA pacing, as described above, is inconclusive.

#### 3.1.6 Bachmann bundle pacing

At the beginning of the new millennium, it was already discussed whether BB pacing is a better alternative to conventional RAA pacing (34). The underlying idea is the promotion of excitation conduction via the BB to shorten interatrial conduction and consequently achieve atrial synchronicity and lower total atrial activation time. These would be optimal baseline conditions to prevent AF. For this reason, it is also reasonable to assume that BB pacing might be particularly beneficial in patients with interatrial blocks and delays (35).

BB pacing results in significantly lower P-wave duration compared with RAA pacing (36) (Figure 3). Furthermore, BB pacing restore atrial synchrony and often provides a more physiological atrial contraction sequence (37). BB pacing thus appears to be the superior pacing sites within the atria that not only positively affects atrial mechanical function but also best fulfils atrial resynchronization function, thereby allowing physiological pacing, particularly in patients with pre-existing interatrial conduction delay (16).

Moreover, the differences between atrioventricular conduction time during atrial pacing are significantly shorter in patients with BB pacing than in patients with RAA pacing (36). In addition, there is evidence that BB pacing may decrease the percentage of ventricular pacing in patients with SND and DDD pacing, providing an additional prevention benefit of AF, because high ventricular pacing percentage is known to be associated with an increased risk of AF (36). Furthermore, BB stimulation seems to be beneficial as it positively affects the mechanical function of the atrium (16).

In the past, experimental animal studies in goats have revealed that stimulation at the BB can prevent, but not completely abolish, the triggering of AF by single premature beats (38). BB pacing thus provides a shortening of the window of inducibility of AF. In these experiments, it was also demonstrated that prevention of AF by pacing relies on prolongation of the premature interval at the BB, effectively preventing conduction block and re-entry (38). It was concluded that the optimal location for preventive stimulation is near the BB and far from the origin of premature beats (38). Similarly, electrophysiological studies in humans indicated that the risk of AF initiation by extrastimuli is significantly decreased with BB pacing compared with conventional RAA pacing (17). BB pacing and RAA pacing were compared in 14 patients undergoing programmed electrical stimulation of the RAA (39). In five patients with RAA pacing, AF was triggered by a critically timed RAA extrastimulus (39). In contrast, AF was not induced in any patient when the RAA extrastimulus was delivered during BB pacing (39). The duration of the P-wave during BB pacing was significantly shorter than that of RAA pacing and sinus rhythm (39). The intraatrial conduction time to the distal coronary sinus caused by an early extrastimulus at the RAA was significantly reduced by BB pacing (39).

In 2001 Bailin et al. performed a multicenter randomized prospective study comparing BB pacing with RAA pacing and the association on recurrent AF in patients with paroxysmal AF (34). A total of 120 patients were included and randomized to BB pacing (*n* = 63) or RAA pacing (*n* = 57) groups. The implantation time of the atrial lead was similar in both groups. No differences in pacing threshold, impedance, or sensing were observed between the BB and RAA groups at implantation or after follow-up periods of 6 weeks, 6 months, and 1 year (34). The percentage of atrial pacing was comparable. Overall P-wave duration was shorter with BB pacing than with RAA pacing. Interestingly, BB atrial pacing even significantly shortened P-wave duration compared with sinus rhythm. In contrast, P-wave duration was longer with atrial pacing from the RAA site compared with sinus rhythm (34). Patients with BB pacing had a significantly higher survival rate without chronic AF (75%) compared with patients with RAA pacing (47%) after 1 year of follow-up. Thus, it could be concluded that BB pacing is not only safe and feasible but, most importantly, can effectively prevent the progression of AF (34).

Recently, Infeld et al. analyzed the atrial arrhythmia burden, recurrence, and new-onset incidence in 241 patients with BB, high IAS, and RAA pacing (11). All patients already presented interatrial conduction delay at the time of implantation and had an atrial pacing percentage of at least 20% (11). It should be emphasized that this is the first study that could reliably define BB pacing by electrocardiographic P-wave and fluoroscopic criteria. This also allowed a reliable differentiation from high IAS pacing (11). Compared with high IAS and RAA pacing, atrial arrhythmia burden was significantly lower in the BB pacing group. Whereas during the 2-year follow-up, atrial arrhythmia burden increased in the low IAS and RAA pacing groups, there was no significant change in the BB pacing group (11). The risk of atrial arrhythmia recurrence was lower in patients with BB pacing than in patients with high IAS and RAA pacing. The risk of new-onset AF was also lower in patients with BB pacing than in patients with high IAS and RAA pacing (11). Infeld et al. addressed in a case series of 24 patients in depth the definition of BB pacing and the optimal placement method to achieve maximum precision (40). According to the authors, pacing at the site where endocardial local BB area potentials are recorded results in a BB pacing P-wave morphology that recapitulates normal sinus P-wave morphology and axis and corrects for baseline interatrial conduction delay (40). This was the first description of the use of local electrograms with paced P-wave morphology to define BB pacing (40). Thus, the right atrial endocardial signature of the BB area may help in the placement of atrial electrodes to find the correct position where pacing will result in atrial activation similar or identical to normal sinus node activation (40).

Recently, van Schie et al. published an analysis of 34 patients undergoing cardiac surgery, during which high-resolution epicardial mapping from the BB was performed both during sinus rhythm and during programmed electrical stimulation (41). Stimulation was performed from the RAA, from the inferior right atrium, meaning the junction of the right atrium with the inferior vena cava, and from the left atrial appendage (41). A reduction in both conduction disturbances and total atrial activation time was most frequently achieved with pacing from the inferior right atrium, especially in patients who already had conduction delay (41). This proof-of-concept study advocates an individualized approach to electrode placement for atrial pacing. Because the optimal pacing site varies interindividual, individualized BB mapping-guided electrode placement for atrial pacing may represent a new opportunity for atrial lead implantation (41).

To summarize, there is evidence that right atrial pacing at the BB has significant beneficial effects on both hemodynamics and the occurrence of AF compared with other pacing locations (35). However, to date, large prospective studies are needed to assess the clinical benefit.

#### 3.1.7 Multisite atrial pacing and biatrial pacing

In biatrial pacing, one pacing lead is located in the right atrium and the other in the coronary sinus, whereas multisite pacing implies at least two pacing sites in the right atrium (42). Multisite atrial pacing has been developed to correct the abnormal atrial activation caused by intra- or interatrial conduction disturbances or by unilateral atrial pacing, because these conditions may promote refractory atrial arrhythmias (43). Left atrial function may be improved by dual right atrial pacing (44). Among other findings, it has been shown that the mean peak transmitral A-wave flow velocity increases under dual-site right atrial pacing compared with baseline, whereas the mean left atrial diameter decreases (44). Dual-site atrial pacing could thus induce long-term atrial reverse remodeling.

Furthermore, it has previously been shown that left atrial contractility was greatest during biatrial pacing (IAS and distal coronary sinus pacing) compared with RAA, IAS, distal coronary sinus, and proximal coronary sinus pacing (45). Biatrial pacing also leads to an increase in cardiac output and a concomitant reduction in postcapillary wedge pressure (46–48). Moreover, biatrial pacing results in left atrioventricular synchrony (45).

Biatrial stimulation can also be referred to as atrial resynchronization therapy (49). A case study by Eicher et al. aimed to explore the role of interatrial dyssynchrony in patients with heart failure with preserved ejection fraction (HFPEF) (50). Findings included a restrictive mitral Doppler pattern, high E/A and E/e' ratios, short A wave duration, increased LA volume with severely depressed function, and severe post-capillary pulmonary hypertension (50). Moreover, electrophysiological studies in these HFpEF patients revealed an interatrial conduction delay, which could be reduced by left atrial pacing through the coronary sinus (50). The study concluded that some HFPEF patients exhibit interatrial conduction delay, delayed left atrial systole, shortened LA emptying, decreased LA compliance, and increased filling pressures (50). The potential benefit of atrial resynchronization therapy in these patients warrants further investigation (49, 50).

There is evidence that multisite and biatrial pacing may be beneficial in selected patient populations, e.g., in recurrent AF or in HFpEF (50, 51). This is reflected in improved electrophysiological and hemodynamic as well as inducibility of AF (17). However, there has been no major randomized-controlled trial of multisite and biatrial pacing, resulting in a very weak evidence base.

Overall, multisite and biatrial pacing are promising techniques to improve atrial hemodynamics and have been suggested to reduce AF burden in previous studies. Because the overall results were not convincing, and the fact that implantation of an additional lead is generally associated with an increased rate of displacement or other complications, these techniques have not been adopted in routine clinical practice.

### 3.2 Atrial pacing modes and atrial fibrillation

In the past, different modes of programming have been evaluated to prevent the occurrence of AF by additional pacing. These are based on two different principles: On the one hand, there is atrial overdrive pacing, which aims to suppress AF by reducing the occurrence of AF episodes. On the other hand, there is reactive antitachycardia pacing (ATP), which aims to reduce the duration of AF and thus the progression of AF (Table 2).

**Table 2:** Atrial pacing algorithms.

	Aim	Randomized-controlled trials
Atrial overdrive pacing	Suppressing atrial fibrillation by reducing the occurrence of atrial fibrillation episodes	ASSERT: algorithm not effective, not well tolerated by patients, faster battery discharging
Antitachycardia pacing (ATP)	Reducing the duration of atrial fibrillation and the progression of atrial fibrillation	MINERVA: ATP effective in reducing the incidence of persistent and permanent atrial fibrillation, decreases risk of early-recurrent atrial fibrillation episodes, promotes left atrial diameter reduction

The ASSERT trial analysed 2,343 patients which were randomized 3 months after pacemaker implantation to have continuous atrial overdrive pacing turned “ON” or “OFF” (52). It has been demonstrated that continuous atrial overdrive pacing does not prevent the occurrence of AF, is not well tolerated by patients, and accelerates pulse generator battery depletion (52). Other prospective studies also failed to demonstrate a significant benefit of atrial overdrive pacing. Neither the occurrence of AF episodes nor the progression to persistent AF could be suppressed (32, 53, 54).

The prospective randomized-controlled MINERVA trial evaluated whether atrial ATP and managed ventricular pacing (MVP) are more effective in preventing progression to permanent AF compared with conventional dual-chamber pacing (DDDR) (55). In this study, it has been revealed that preventive atrial pacing and atrial ATP (DDDRP) in combination with MVP reduced the incidence of permanent AF compared with standard dual-chamber pacing (DDDR). A substudy of the MINERVA trial identified high reactive ATP efficacy as an independent predictor of lower risk for permanent or persistent AF (56). Furthermore, the MINERVA study addressed the influence of atrial ATP on atrial remodeling induced by atrial arrhythmias (57). Remodeling was measured by the early recurrence of atrial arrhythmias and the change in left atrial diameter. Accordingly, electrical remodeling of the atrium becomes apparent after approximately 12 h of continuous arrhythmia (57). Compared with DDDRP or MVP, DDDRP + MVP decreases early recurrent AF and promotes left atrial diameter reduction, suggesting that atrial ATP may reverse electrical and mechanical remodeling (57). A few years later, a comparative non-randomized evaluation of the MINERVA trial was performed to determine whether reactive ATP may be the primary cause of reduction in persistent or permanent AF independent of preventive pacing (58). Therein, the use of reactive ATP was associated with a lower incidence of persistent AF, highlighting that the positive results of the MINERVA trial were related to the efficacy of reactive ATP rather than to preventive pacing (58).

The specific atrial pacing algorithms have been steadily improved over several generations in recent years. Atrial overdrive pacing, which aims to reduce and suppress AF episodes, has not demonstrated a benefit, and is not well tolerated by patients, whereas reactive ATP may effectively terminate AF episodes, thereby reducing the progression of AF.

## 4 Ventricular pacing

### 4.1 Ventricular pacing and the risk of atrial fibrillation

Ventricular pacing has a strong association with an increased risk of AF (59–62). This risk may be as high as 24% within 1 year after pacemaker implantation (63). In particular, high cumulative ventricular pacing of ≥50% is associated with an increased likelihood of developing atrial high-rate episodes and AF (63, 64). This results in the recommendation to avoid unnecessary right ventricular stimulation whenever possible (2, 63, 64). Rationale for the increased likelihood of AF after pacemaker implantation with ventricular pacing may be that chronic right ventricular pacing leads to atrial remodeling, electrical as well as structural, and decreased atrial function (6, 9, 65). Concomitantly, this is also associated with increased filling pressures, leading to mitral regurgitation and pulmonary vein distention, and also compromising left ventricular systolic function (10, 65, 66).

### 4.2 Atrial vs. ventricular pacing

In the past, atrial pacing has been demonstrated to be associated with significantly higher survival, and concomitantly less AF and fewer thromboembolic complications (61). In patients with SND, VVI pacing significantly increases AF and mortality compared with AAI pacing (1). SND *per se* is already an independent risk factor for the development of AF. Therefore, in patients with SND, AAI(R) should be the preferred pacing mode, and in other patients without chronic AF, DDD(R) should be used to prevent AF (1). However, there are other recommendations. In the randomized-controlled DANPACE trial, 1,415 patients with SND with single-chamber atrial pacing (AAIR) or dual-chamber pacing (DDDR) were compared (67). Paroxysmal AF was observed in 201 patients (28.4%) in the AAIR group vs. 163 patients (23.0%) in the DDDR group, which indicated a significant difference. In contrast, the incidence of chronic AF as well as stroke did not differ between treatment groups. Therefore, the authors argue for a preference of DDDR pacemakers over AAIR pacemakers in patients with SND (67).

### 4.3 Single-camber vs. dual-chamber pacemakers

In 2002, the MOST trial analyzed 2,010 patients with SND assigned to dual-chamber pacing or ventricular pacing (62). The primary end point was death from any cause or nonfatal stroke. Secondary end points included the combination of death, stroke, or hospitalization for heart failure, AF, heart failure score, pacemaker syndrome, and quality of life (62). After a mean follow-up of 33 months, the incidence of the primary endpoint was not significantly different between the dual-chamber group (21.5%) and the ventricular pacing group (23.0%) (62). However, the dual-chamber pacing group had a lower risk of AF (62).

Similarly, a substudy of the PASE trial concluded that a DDD pacemaker was superior to ventricular-only pacing. In a study cohort of 407 patients aged at least 65 years, the VVIR mode was independently associated with a 2.6-fold increased relative risk of developing AF after pacemaker implantation compared with the DDDR mode in patients with SND (68). Thus, in elderly patients with SND requiring permanent pacing, the DDDR pacing mode was protective against the development of AF (68).

Nevertheless, there were also controversial findings that did not demonstrate superiority of DDD pacing compared with VVI pacing (69). In the UKPACE trial, 2,021 patients older than 70 years with atrioventricular block and either single-chamber or dual-chamber pacing were studied (70). No significant differences were found between the single-chamber pacing group and the dual-chamber pacing group in the incidence of AF, heart failure, or a combination of stroke, transient ischemic attack, or other thromboembolism after an observation period of 3 years (70). Furthermore, the risk of AF increased linearly with the cumulative percentage of pacing from 0% to 85% both in the DDD and VVI pacemakers (59).

Consequently, it is important to minimize the ventricular pacing burden, which can be achieved both by programming the atrioventricular interval and by using specific algorithms to minimize right ventricular pacing (2). Adjusting the atrioventricular interval, for instance, by repetitive atrioventricular hysteresis or atrioventricular search hysteresis (e.g., AV Search+, dynamic AV delay), can prevent unnecessary ventricular pacing (2, 6). Improved AV timing enhances diastolic filling by allowing optimal preload and efficient ventricular filling. Optimal AV delay improves E- and A-wave integration in the mitral inflow profile, reducing diastolic mitral regurgitation and enhancing effective LV filling (71). Premature ventricular contraction (short AV delay) can decrease filling time and thus reduce stroke volume and cardiac output (71). Delayed ventricular contraction (long AV delay), on the other hand, can lead to an inadequate contribution of atrial contraction, increasing left atrial pressure and potentially causing atrial enlargement and AF (71). Furthermore, it was demonstrated that restoring AV coupling via biventricular pacing significantly improves haemodynamics in both normal and failing hearts with prolonged AV conduction (71). The combination of optimizing AV timing and considering biventricular or left-only ventricular pacing with cardiac resynchronization therapy offers a differentiated, individualized approach to improving cardiac function in patients with heart failure and may also contribute to the prevention of AF through improved hemodynamics (71–73).

Sweeney et al. evaluated the benefit of minimal ventricular pacing in the SAFE-PACe trial of 1,065 patients with SND assigned to DDD or DDD-minimal ventricular pacing (60). Persistent AF developed in 12.7% in the conventional dual-chamber pacing group and 7.9% in the dual-chamber minimal ventricular pacing group (60). The hazard ratio for the development of persistent AF in patients with dual-chamber minimal ventricular pacing compared with those with conventional dual-chamber pacing was 0.60, which represented a 40% reduction in relative risk (60). The authors concluded that compared with conventional dual-chamber pacing, dual-chamber minimal ventricular pacing can prevent ventricular desynchronization and moderately reduce the risk of persistent AF in patients with SND and is thus superior to conventional dual-chamber pacing in preventing the progression of AF (60). Additionally, a substudy of the MINERVA trial investigated the benefit of managed ventricular pacing regarding the risk of AF (74). 1,166 patients were randomized to either DDDRP control, MVP (managed ventricular pacing), or atrial ATP plus MVP (DDDRP + MVP), and the interaction of PR interval with pacing mode was examined by comparing the risk of AF for more than seven consecutive days (74). The risk of persistent AF was lower in patients with short PR interval (shorter than 180 ms, the median PR interval) when programmed in MVP mode than in patients with long PR interval (equal to or longer than 180 ms) when programmed in DDDR mode than in MVP (74). This suggests that the PR interval can be used as a selection criterion for determining the optimal physiological pacing mode (74). The incidence of persistent AF was lower in patients with short PR interval treated with minimization of right ventricular pacing than in patients with long PR treated with standard dual-chamber pacing (74).

### 4.4 Different ventricular pacing sites

Historically, the right ventricular apex was considered the standard implantation site for the ventricular lead, and it remains a preferred implantation site in many centres. However, there have been efforts in recent years to adapt the ventricular implantation site to a more physiological excitation and contraction to achieve synchrony of the two ventricles and thereby improved hemodynamics. Alternative implantation sites represent parahisic and His-bundle pacing, septal pacing, and left bundle branch pacing (Figure 1 right, Figures 2, 3) (Table 3).

**Table 3:** Different ventricular pacing sites and their advantages and disadvantages.

Ventricular pacing site	Advantages	Disadvantages
Right ventricular apex	• Simple, even easily achievable for the inexperienced	• Evidence for increased AF risk, especially in a high pacing burden • Unphysiological stimulation and excitation propagation
High septal	• Relatively easy to position • More physiological excitation propagation • Particularly low risk of perforation	• Higher rate of lead displacement • In studies no clear advantage compared to the apical position
His bundle	• Evidence for lower risk of new-onset AF, persistent and permanent AF	• No benefit in prevention of AF progression • More complex positioning by means of navigable sheath and electrophysiological localization • Higher rate of lead displacements • Increase in the threshold of stimulation in the medium term
Left bundle branch	• Evidence for lower risk of new-onset AF and AF burden	• More complex positioning by means of a special sheath • Higher rate of lead displacements • Limited experience to date in the long-term

#### 4.4.1 High right ventricular septal pacing

High right ventricular septal pacing is an alternative to apical pacing. However, regarding the occurrence of pacemaker-induced AF, the evidence is very limited. The Protect-Pace trial studied 240 patients with high-grade atrioventricular block who required at least 90% ventricular pacing burden and were randomized to right ventricular apical or high-septal pacing groups (75). After a median follow-up of 2 years, there was no difference in AF burden between the two groups (75). Accordingly, superiority of high-septal right ventricular pacing over conventional pacing could not be demonstrated.

#### 4.4.2 His-bundle pacing

Hisian area pacing appears to be associated with a lower risk of persistent and permanent AF compared with right ventricular apical or right ventricular septal pacing (76, 77). Similarly, when compared with standard DDD pacemaker with an algorithm to avoid unnecessary right ventricular pacing, persistent AF occurred significantly less frequently with His bundle pacing (78). Recently, a study by Ravi et al. demonstrated that in patients without a history of AF, His-bundle pacing had a lower risk of new-onset AF compared with standard right ventricular pacing (79). This benefit remained significant at different pacing burdens and was observed at burdens greater than 20%, ≥40%, ≥60%, and ≥80% (79). However, there was no difference at pacing burden <20%. There was also no difference in AF progression in patients with a history of AF (79). Thus, overall, compared with standard right ventricular pacing, His-bundle pacing had a lower risk of new-onset AF, which was particularly observed at a higher pacing burden (79). These findings were also recently confirmed by a meta-analysis: Compared with standard right ventricular pacing, his bundle pacing was associated with a lower risk of new-onset AF (RR 0.61) (80).

#### 4.4.3 Left bundle branch area pacing

There is also preliminary evidence with left bundle branch pacing that it may be beneficial in the prevention of AF. Recently, left bundle branch area pacing was shown to be associated with a significantly lower risk of new diagnosis of AF ≥30 s and >6 min compared with standard right ventricular pacing (81). However, comparable to other studies, there was no difference in patients with a ventricular pacing burden <20%, suggesting that patients with a higher ventricular pacing burden are more likely to benefit from left bundle branch area pacing to prevent AF (81). Zhu et al. examined the risk of new-onset AF in 527 patients with left bundle branch area pacing compared with right ventricular pacing (82). During a mean follow-up of 11.1 months, left bundle branch area pacing resulted in a significantly lower incidence of new-onset AF and lower AF burden than right ventricular pacing (82). In patients with a ventricular pacing burden ≥20%, left bundle branch area pacing was associated with a lower risk of new-onset AF compared with right ventricular pacing, although the effect of pacing modality was no longer evident in patients with a ventricular pacing burden <20% (82). Therefore, they also concluded that left bundle branch area pacing has a lower risk of AF occurrence compared with right ventricular pacing and that patients with a high ventricular pacing burden may in particular benefit from left bundle branch area pacing (82).

## 5 Approaches for the prevention of atrial fibrillation in patients with pacemaker indication

**Figure 4:**
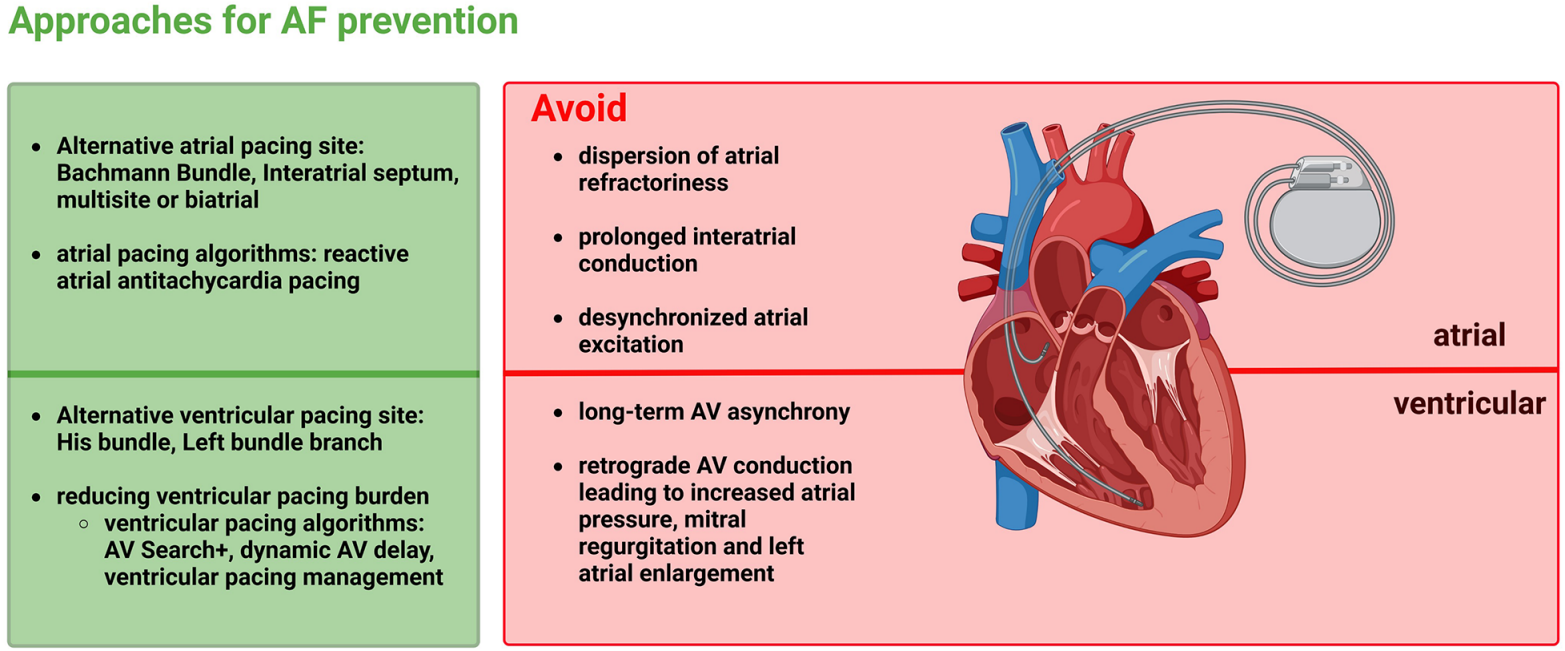
Approaches for atrial fibrillation prevention by minimizing the adverse effects of atrial as well as ventricular pacing.

As described above, AF occurs frequently in patients with SND and interatrial conduction delay. This may be explained in part by the fact that atrial ectopy is more likely to occur in these conditions, which itself may trigger AF. For this reason, it is useful to analyse pacing strategies that could help reduce the likelihood of new AF occurrence and prevent AF progression. AF-promoting atrial factors include dispersion of atrial refractoriness, prolonged interatrial conduction, and desynchronized atrial excitation. For this reason, it seems feasible that atrial pacing is optimized when dispersion of atrial refractoriness is as low as possible, interatrial excitation conduction is as fast as possible, and atrial activation is maximally synchronized to minimize the risk for AF. Ventricular factors promoting AF include pacing-triggered desynchrony of the two ventricles, a high ventricular pacing rate, and ventricular pacing-triggered increased mitral regurgitation and increased atrial filling pressures, which may cause pulmonary vein reflux and dilatation, thereby triggering AF. This implies that there are prevention approaches for AF at the atrial as well as ventricular level (Figure 4).

At the atrial level, the atrial pacing site and specific atrial pacing algorithms represent adjusting screws for prevention of AF. The present review elaborated that the RAA, as a standard atrial pacing site, is inferior to other pacing sites both electrophysiological and hemodynamically and also in the prevention of AF (11, 20, 21). IAS pacing, BB pacing, and biatrial or multi-site atrial pacing represent alternative pacing sites that may be more beneficial for the prevention of AF. These alternative pacing sites should be considered especially in patients at increased risk for AF, e.g., with pre-existing interatrial conduction delays and interatrial blocks, but, on the other hand, also in pacemaker patients who are at a younger age and, accordingly, will have to live with a pacemaker for a long time. Patients who are expected to have a high atrial pacing rate could also benefit from an alternative pacing site. Even those patients already suffering from AF may benefit from an alternative pacing site, as the progression of AF can be prevented, and AF burden can be reduced (21, 27, 29). BB pacing has been particularly beneficial and may have the greatest potential of the various atrial pacing sites in the prevention of AF (11, 34).

Specific atrial pacing algorithms might represent another possibility for the prevention of AF. Whereas atrial overdrive pacing, which aims to reduce and suppress AF episodes, has failed to demonstrate benefit, reactive ATP may be effective in terminating AF duration and thus significantly reducing AF progression (55, 56, 83). Therefore, atrial ATP presents an option for prevention of longer-lasting AF, particularly in patients with pre-existing paroxysmal AF. In contrast, the benefit of atrial overdrive pacing and ATP in the prevention of new-onset AF must be considered rather critically (52, 83).

Alternative pacing sites should also be considered at the ventricular level to prevent AF. To date, it is well known that ventricular pacing increases the risk of AF (63). Pacing in the right ventricular apex is still often the standard pacing site in many centres, but alternative septal pacing sites such as His bundle and especially left bundle branch pacing have become more popular in recent years. There are first data showing that prevention of AF may be more successful compared to the conventional apical pacing site (76, 79, 81, 82). In the next few years, studies will certainly be initiated to investigate the incidence or recurrence of AF as well as its progression in more detail.

Another important approach is the reduction of ventricular pacing burden. A high ventricular pacing burden increases the risk for AF (63, 64). For this reason, DDD pacing and MVP may be beneficial and should be preferred to ventricular pacing alone in appropriate patients (74). This also indicates that in patients who are expected to have a high pacing burden, they may benefit from a ventricular septal pacing site such as left bundle branch pacing.

Nevertheless, it must be mentioned that current large randomized-controlled studies concerning the prevention of pacemaker-induced AF are limited. In particular, with regard to atrial pacing, there were several studies around the turn of the millennium that questioned the RAA as a pacing site and demonstrated an advantage of alternative pacing sites. However, this has not been universally established in routine clinical practice and it has not been consistently followed for longer, despite clear data that IAS pacing and especially BB pacing have advantages in the prevention of AF. In the last 20 years, there have been many changes in the pacing management of patients, both device-related but also in the demographic characteristics and general cardiac management of patients. Therefore, there is a need for randomized-controlled trials to help selecting which patient would benefit from which pacing site and mode, atrial as well as ventricular. An individualized approach might be favoured.

## 6 Conclusions

AF is common in pacemaker patients and in patients with SND in general. The present review outlined that there are both atrial and ventricular pacing adjustments, mainly involving pacing sites and pacing modes, to prevent AF. IAS pacing and particularly BB pacing are preferable to conventional RAA pacing, especially in patients with pre-existing interatrial conduction delay. In patients with a history of AF, atrial ATP may prevent AF progression. A further highly important target in the prevention of AF is the reduction of ventricular pacing burden. In addition, there is preliminary evidence that alternative ventricular pacing sites such as His bundle and left bundle branch pacing may be superior to conventional right ventricular apical pacing in the prevention of AF.
